# Nitric Oxide Signalling in Descending Vasa Recta after Hypoxia/Re-Oxygenation

**DOI:** 10.3390/ijms23137016

**Published:** 2022-06-24

**Authors:** Minze Xu, Falk-Bach Lichtenberger, Cem Erdoǧan, Enyin Lai, Pontus B. Persson, Andreas Patzak, Pratik H. Khedkar

**Affiliations:** 1 Institute of Translational Physiology, Charité—Universitätsmedizin Berlin, Corporate Member of Freie Universität Berlin and Humboldt-Universität zu Berlin, Charitéplatz 1, 10117 Berlin, Germany; minze.xu@charite.de (M.X.); falk.lichtenberger@charite.de (F.-B.L.); cem.erdogan@charite.de (C.E.); pontus.persson@charite.de (P.B.P.); pratik.khedkar@charite.de (P.H.K.); 2 Department of Physiology, Zhejiang University School of Medicine, Hangzhou 310058, China; laienyin@zju.edu.cn

**Keywords:** descending vasa recta, hypoxia, re-oxygenation, soluble guanylyl cyclase, nitric oxide

## Abstract

Reduced renal medullary oxygen supply is a key factor in the pathogenesis of acute kidney injury (AKI). As the medulla exclusively receives blood through descending vasa recta (DVR), dilating these microvessels after AKI may help in renoprotection by restoring renal medullary blood flow. We stimulated the NO-sGC-cGMP signalling pathway in DVR at three different levels before and after hypoxia/re-oxygenation (H/R). Rat DVR were isolated and perfused under isobaric conditions. The phosphodiesterase 5 (PDE5) inhibitor sildenafil (10^−6^ mol/L) impaired cGMP degradation and dilated DVR pre-constricted with angiotensin II (Ang II, 10^−6^ mol/L). Dilations by the soluble guanylyl cyclase (sGC) activator BAY 60-2770 as well as the nitric oxide donor sodium nitroprusside (SNP, 10^−3^ mol/L) were equally effective. Hypoxia (0.1% O_2_) augmented DVR constriction by Ang II, thus potentially aggravating tissue hypoxia. H/R left DVR unresponsive to sildenafil, yet sGC activation by BAY 60-2770 effectively dilated DVR. Dilation to SNP under H/R is delayed. In conclusion, H/R renders PDE5 inhibition ineffective in dilating the crucial vessels supplying the area at risk for hypoxic damage. Stimulating sGC appears to be the most effective in restoring renal medullary blood flow after H/R and may prove to be the best target for maintaining oxygenation to this vulnerable area of the kidney.

## 1. Introduction

Despite its energy-intensive functions of resorption and concentration, the renal medulla receives a considerably small amount (~10%) of the renal blood flow (RBF). It is exclusively perfused through descending vasa recta (DVR), which are capillary-like, long vessels originating from the juxtamedullary nephrons. DVR are lined with pericytes on their outer surface [1,2,3]. DVR supply the most energy-consuming cells, while warranting minimum perfusion to glycolytic cells in remote regions of the inner medulla. Highly oxygen-consuming structures, such as the S3 segment and the thick ascending limb of Henle, rely on vasa recta anastomosis for their oxygen supply. On the other hand, blood supply to deeper structures in the papilla must be minimal to prevent osmolyte washout. High oxygen demand combined with low perfusion renders the inner part of the outer medulla particularly susceptible to hypoxic damage in pathological events such as acute kidney injury (AKI) and chronic kidney disease (CKD) [4]. As DVR are the only vessels supplying blood to these renal areas at risk of hypoxia, maintaining blood supply is uniquely demanding and is key to providing renoprotection.

The physiological functions of DVR have been extensively characterized over the last two decades and the specific role that pericytes play in this context has also been described (for review see [5,6,7]). It is the response of pericytes to various stimuli, such as sympathetic nervous activity, circulating and local hormones, and metabolites generated by neighbouring tubuli, that enables DVR to constrict and relax [5,8,9].

While experimental ischemia/re-perfusion reduces the overall RBF, its effect on the blood flow and oxygenation of the medulla is significantly prolonged compared to the cortex [10,11]. Moreover, the damage is especially severe in the inner part of the outer medulla [12]. These findings suggest that medullary blood flow plays an important role in the genesis of renal damage in various renal pathologies. RBF is critically regulated by nitric oxide (NO) through its vasodilatory effect. NO is probably the strongest antagonist of several vasoconstrictors, including angiotensin II (Ang II), and plays an important role in the physiology and pathophysiology of renal perfusion [13,14,15,16]. Experimentally induced NO deficiency has been shown to reduce RBF and cause renal damage in several species [17,18]. NO deficiency is a hallmark of AKI and CKD and may contribute to the imbalance of vasoconstrictor and dilator mechanisms that increase renal vascular resistance and reduce cortical and medullary RBF [19]. Therefore, improving RBF, especially the medullary flow, by restoring NO production and signalling may be a protective and therapeutic tool in AKI and CKD. Pharmacological approaches have indeed been successful in animal experiments; however, they have not been translated to the clinical setting to date.

Cyclic GMP (cGMP) is the mediator of the NO system in vascular smooth muscle cells. Several pharmacological agents developed during the last two decades aim at modulating the effects of the NO system by increasing cellular levels of cGMP. The most prominent categories of such agents are phosphodiesterase 5 (PDE5) inhibitors and soluble guanylyl cyclase (sGC) stimulators and activators [20,21]. Some of them are already used in the clinic, e.g., to treat pulmonary hypertension [22]. Their dilatory capabilities in microvessels of the renal cortex have recently been demonstrated [23]. Interestingly, the sGC activator cinaciguat has been shown to dilate glomerular efferent but not afferent arterioles in mice after strong hypoxia and subsequent re-oxygenation [24]. This indicates that NO signalling after hypoxia is differently regulated in the two different types of glomerular arterioles. However, little is known about the influence of hypoxia on microvascular NO signalling in the renal medulla. Therefore, we investigated the dilatory capacity of the NO system in DVR and tested the ability of an sGC activator to dilate these microvessels after hypoxia/re-oxygenation (H/R). This could provide a new approach for protection and therapy in AKI and CKD.

## 2. Results

### 2.1. Pharmacological Characterization of NO-sGC-System in Rat DVR

#### 2.1.1. Effect of NOS Inhibition

**Figure 1:**
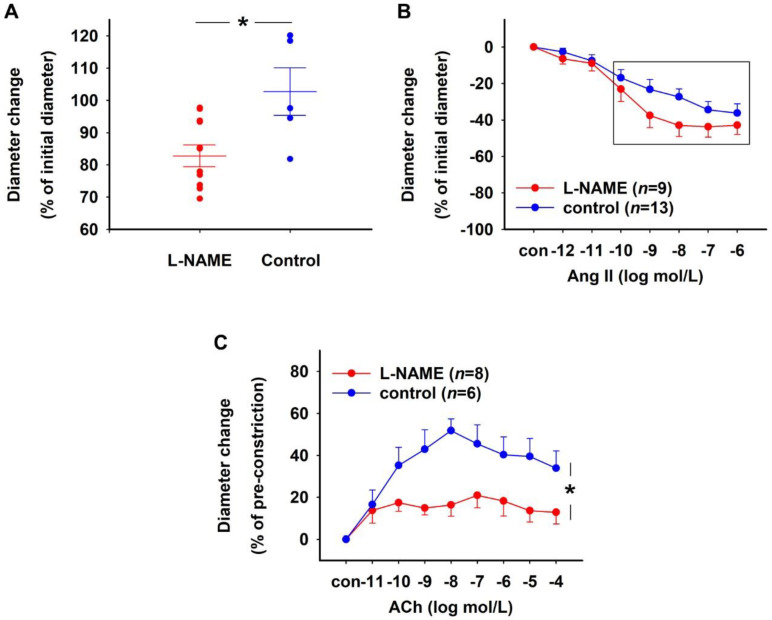
Effect of L-NAME on rat DVR. (**A**) Vessels treated with L-NAME (10^−4^ mol/L) for 15 min (*n* = 12) showed significant constriction compared to untreated control (*n* = 5) vessels (Mann–Whitney test, * *p* < 0.05). Vessel diameters in both groups before treatment were not significantly different (mean ± SEM: 8.48 ± 0.84 µm (L-NAME) vs. 7.84 ± 0.83 µm (control), Mann–Whitney test, *p* > 0.05). (**B**) Concentration–response curves showing the constriction induced by Ang II in DVR with and without 10^−4^ mol/L L-NAME pre-treatment for 15 min. L-NAME-treated DVR constricted significantly more in response to higher Ang II concentrations (highlighted in a box) than the control group (Brunner test, * *p* < 0.001). (**C**) Concentration–response curves showing the relaxation induced by 10^−11^–10^−4^ mol/L acetylcholine (ACh) in DVR with and without pre-treatment with 10^−4^ mol/L L-NAME for 15 min. L-NAME-treated vessels showed a lower maximum response to ACh compared to the control group (Brunner test, * *p* < 0.01).

NO deficiency was induced in isolated rat DVR by treating them with an inhibitor of NO synthases (NOS)—Nω-nitro-L-arginine methyl ester hydrochloride (L-NAME, 10^−4^ mol/L)—for 15 min. While a control group of vessels incubated under similar conditions did not show a significant change in diameter, vessels treated with L-NAME were significantly constricted (Figure 1A).

L-NAME-treated and untreated control vessels were subjected to increasing concentrations of Ang II (10^−12^–10^−6^ mol/L) to study the effect of NOS inhibition on vasoconstriction. L-NAME-treated vessels showed a significantly stronger constriction in response to higher concentrations of Ang II (10^−10^–10^−6^ mol/L) compared to control vessels (Figure 1B). The absolute initial diameters of L-NAME-treated vessels (mean ± SEM: 7.17 ± 0.99 µm) were similar to control vessels (7.87 ± 0.70 µm, Mann–Whitney test, *p* > 0.05). To assess if NOS inhibition also affects vasodilation, L-NAME-treated and untreated control vessels were pre-constricted using 10^−6^ mol/L Ang II followed by treatment with cumulatively increasing concentrations of acetylcholine (ACh, 10^−11^–10^−4^ mol/L). The dilatory response of L-NAME-treated vessels was, indeed, significantly weaker compared to control vessels (Figure 1C). The absolute initial diameters of the vessels after pre-constriction were not significantly different between the L-NAME-treated and control groups (mean ± SEM: 1.97 ± 0.41 µm (L-NAME) vs. 1.80 ± 0.11 µm (control), Mann–Whitney test, *p* > 0.05).

#### 2.1.2. Effect of PDE5 Inhibition

**Figure 2:**
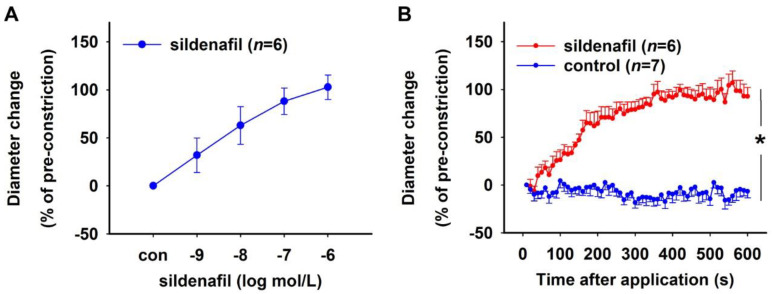
Effect of the PDE5 inhibitor sildenafil. Rat DVR were pre-constricted with 10^−^^6^ mol/L Ang II. (**A**) Concentration–response curve showing vasorelaxation induced by 10^−9^ to 10^−6^ mol/L sildenafil. Vessel diameter did not change in the absence of sildenafil (con). (**B**) Time–response curves showing relaxation induced by 10^–7^ mol/L sildenafil over a period of 10 min. Sildenafil caused an almost instantaneous relaxation of the vessels with 100% relaxation achieved in 5 min (Brunner test, * *p* < 0.001). Control vessels without sildenafil treatment remained constricted throughout the experiment.

Vasodilation was tested by subjecting isolated rat DVR to cumulatively increasing concentrations of sildenafil (10^−9^–10^−6^ mol/L). Vessels were pre-constricted using Ang II (10^−6^ mol/L) and had a mean diameter ± SEM of 3.65 ± 0.32 µm. The pre-constricted vessels showed concentration-dependent dilation in response to sildenafil (Figure 2A). Bolus application of sildenafil (10^−7^ mol/L) to Ang II pre-constricted vessels resulted in 100% dilation of vessels in 5 min, while control vessels, which did not receive a bolus, remained constricted throughout the experimental duration of 10 min (Figure 2B). Both sildenafil-treated and untreated control vessels had comparable absolute diameters after Ang II pre-constriction (mean ± SEM: 3.53 ± 0.44 µm (sildenafil) vs. 3.37 ± 0.47 µm (control), Mann–Whitney test, *p* > 0.05).

#### 2.1.3. Effect of sGC Activation in NO-Deficient Vessels

**Figure 3:**
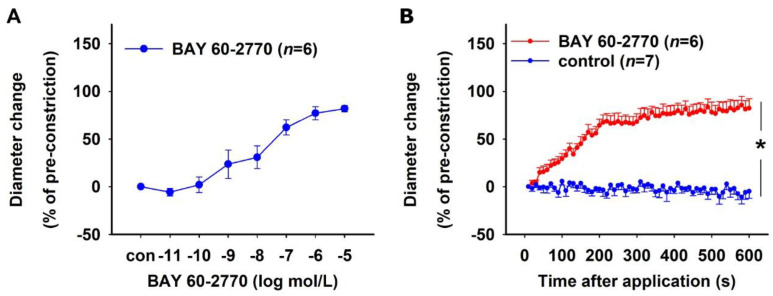
Effect of the soluble guanylyl cyclase activator—BAY 60-2770. Rat DVR were pre-treated with 10^−4^ mol/L L-NAME and pre-constricted with 10^−6^ mol/L Ang II. (**A**) Concentration–response curve showing relaxation induced by 10^−11^–10^−5^ mol/L BAY 60-2770. Vessel diameter did not change in the absence of BAY 60-2770 (con). (**B**) Time–response curves showing relaxation induced by 10^−6^ mol/L BAY 60-2770 over a period of 10 min. Control vessels without BAY 60-2770 treatment remained constricted throughout the experiment (Brunner test, * *p* < 0.001).

sGC was activated using increasing concentrations of the NO-independent activator BAY 60-2770. Rat DVR were pre-treated with 10^−4^ mol/L L-NAME followed by a pre-constriction with 10^−6^ mol/L Ang II. A concentration-dependent dilation was seen in response to BAY 60-2770 (Figure 3A). The absolute initial diameter of vessels after pre-constriction was 3.59 ± 0.32 µm (mean ± SEM). In another set of experiments, L-NAME-treated Ang-II-constricted vessels that received a bolus of 10^−6^ mol/L BAY 60-2770 showed maximum dilation in 6 min, while the vessels that did not receive the bolus remained constricted throughout the duration of the experiment (10 min, Figure 3B). Both groups of vessels had comparable initial diameters after pre-constriction with Ang II (mean ± SEM: 3.22 ± 0.38 µm (BAY 60-2770) vs. 2.90 ± 0.54 µm (control), Mann–Whitney test, *p* > 0.05).

### 2.2. Characterization of Human DVR

**Figure 4:**
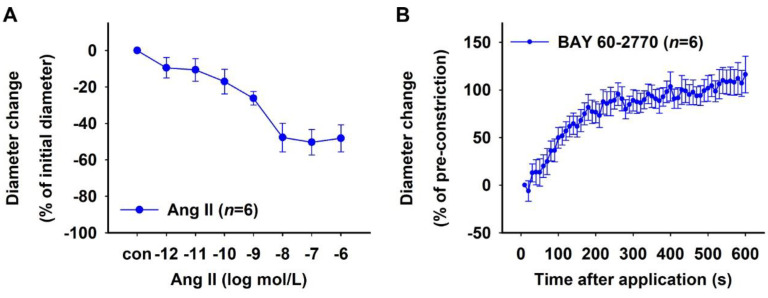
sGC activation in human DVR. (**A**) Concentration–response curve showing constriction induced by 10^−12^–10^−6^ mol/L Ang II. Vessel diameters did not change in the absence of Ang II (con). (**B**) Time–response curve showing relaxation induced by 10^−6^ mol/L BAY 60-2770. Vessels were pre-constricted with 10^−6^ mol/L Ang II. BAY 60-2770 caused an almost instantaneous relaxation of the vessels with maximum relaxation achieved in 3 min.

Human DVR were isolated from tissue samples obtained from nephrectomies. The viability of the vessels was tested by treating them with increasing concentrations of Ang II (10^−12^–10^−6^ mol/L). The vessels constricted in a concentration-dependent fashion in response to Ang II (Figure 4A). The initial absolute diameter of the vessels was 11.29 ± 0.88 µm (mean ± SEM). To test the effect of sGC activation on human DVR, Ang II pre-constricted vessels were treated with a bolus of BAY 60-2770 (10^−6^ mol/L). The vessels achieved maximum relaxation 3 min post bolus application (Figure 4B).

### 2.3. Effect of H/R on Rat DVR

**Figure 5:**
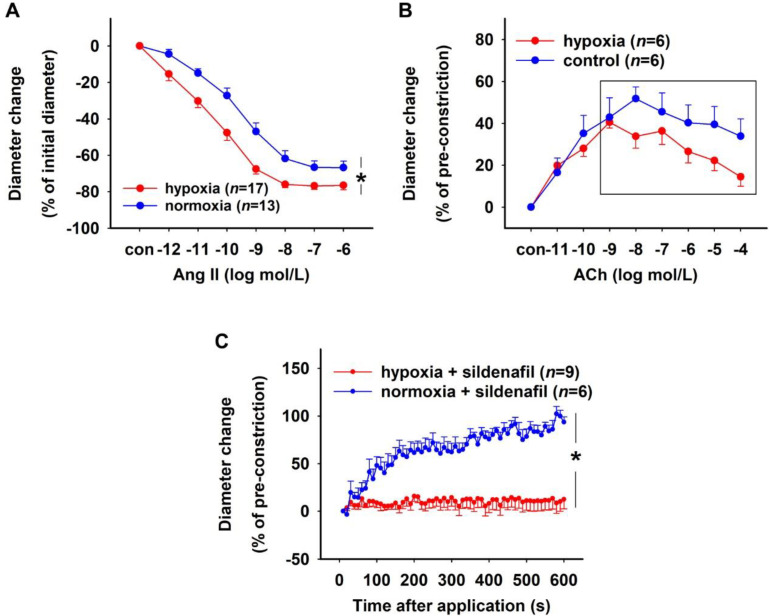
Effect of hypoxia/re-oxygenation on rat DVR. Vessels were pre-incubated either in a 0.1% oxygen (O_2_) atmosphere (hypoxia) or in a 20.9% O_2_ atmosphere (normoxia) for 30 min. (**A**) Concentration–response curves showing constriction induced by 10^−12^–10^−6^ mol/L Ang II in hypoxic and normoxic vessels. Hypoxia/re-oxygenation group of vessels showed a significantly stronger constriction in response to Ang II compared to the normoxia group (Brunner test, * *p* < 0.001). (**B**) Concentration–response curve showing relaxation induced by 10^−11^–10^−4^ mol/L ACh in hypoxic and normoxic vessels pre-constricted with 10^−6^ mol/L Ang II. Hypoxic vessels relaxed significantly less in response to higher concentrations of ACh (10^−9^–10^−4^ mol/L, highlighted in a box) compared to normoxic vessels (Brunner test, * *p* < 0.05). (**C**) Time–response curves showing the effect of PDE inhibition using 10^−6^ mol/L sildenafil on hypoxic and normoxic vessels pre-constricted with 10^−6^ mol/L Ang II. Sildenafil caused normoxic vessels to relax, while no relaxation was observed in hypoxic vessels for the entire duration of the experiment (Brunner test, * *p* < 0.001).

Isolated rat DVR were incubated in a 0.1% O_2_ environment (hypoxia) or a 20.9% O_2_ environment (normoxia) for 30 min. Hypoxia did not affect the resting diameters of the vessels (mean ± SEM: 7.31 ± 0.45 µm (hypoxia) vs. 7.91 ± 0.50 µm (normoxia), Mann–Whitney test, *p* > 0.05). To study the effect of hypoxia on vasoconstriction, both groups of vessels were treated with increasing concentrations of Ang II. Hypoxic vessels showed a significantly stronger constriction in response to Ang II (Figure 5A). The effect of hypoxia on vasodilation was analysed by pre-constricting hypoxic and normoxic vessels with 10^−6^ mol/L Ang II followed by treatment with cumulatively increasing concentrations of ACh. While both groups had similar diameters after pre-constriction (mean ± SEM: 1.48 ± 0.10 µm (hypoxia) vs. 1.97 ± 0.41 µm (control), Mann–Whitney test, *p* > 0.05), hypoxic vessels showed a significantly weaker relaxation in response to ACh compared to normoxic vessels (Figure 5B). In another set of experiments, hypoxic and normoxic vessels pre-constricted using 10^−6^ mol/L Ang II received a bolus of 10^−6^ mol/L sildenafil. While PDE5 inhibition with sildenafil resulted in the complete relaxation of normoxic vessels, hypoxic vessels remained constricted throughout the experimental duration of 10 min (Figure 5C). The initial diameters of hypoxic vessels after pre-constriction (mean ± SEM: 2.65 ± 0.47 µm) were not significantly different compared to normoxic vessels (3.53 ± 0.44 µm, Mann–Whitney test, *p* > 0.05).

**Figure 6:**
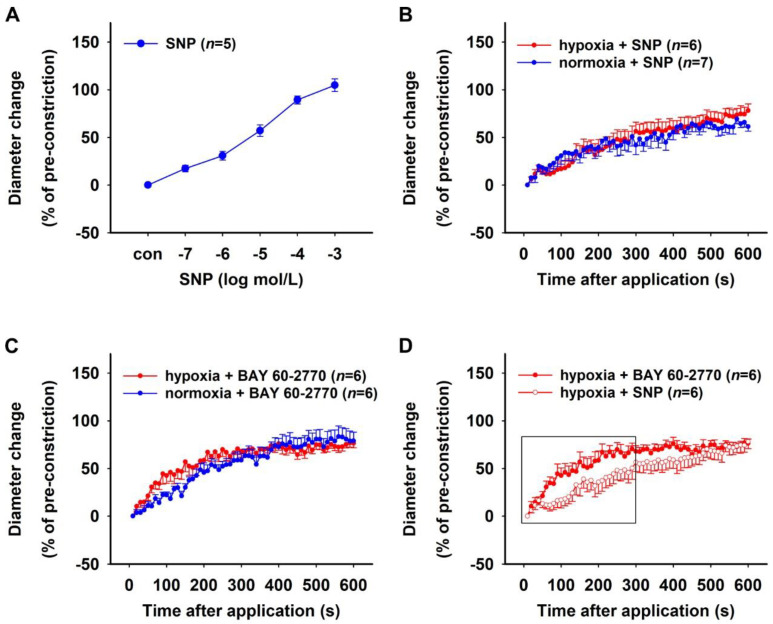
Effects of sodium nitroprusside (SNP) and BAY 60-2770 on hypoxic rat DVR. (**A**) Concentration–response curve showing relaxation induced by 10^−11^–10^−5^ mol/L sodium nitroprusside (SNP) in rat vasa recta. Vessel diameter did not change under control conditions (con), i.e., in the absence of BAY 60-2770. Time–response curves showing the effect of (**B**) the NO donor SNP (10^−3^ mol/L) and (**C**) the NO-independent sGC activator BAY 60-2770 (10^−6^ mol/L) on hypoxic and normoxic vessels over a period of 10 min. Vessels were pre-incubated either in a 0.1% oxygen (O_2_) atmosphere (hypoxia) or in a 20.9% O_2_ atmosphere (normoxia) with 10^−4^ mol/L L-NAME for 30 min followed by a pre-constriction with 10^−6^ mol/L Ang II. Relaxation to SNP and BAY 60-2770 were similar in hypoxic and normoxic vessels, respectively. However, (**D**) hypoxic vessels showed faster relaxation in response to BAY 60-2770 compared to SNP (Brunner test, *p* < 0.05, same data as (**B**,**C**)).

The effect of sGC activation on hypoxic vessels was analysed using the NO donor sodium nitroprusside (SNP) and the NO-independent sGC activator BAY 60-2770. To determine the concentration response, SNP was used in cumulatively increasing concentrations to treat isolated rat DVR that were pre-constricted with 10^−6^ mol/L Ang II and pre- treated with 10^−4^ mol/L L-NAME (mean diameter ± SEM after pre-constriction: 3.59 ± 0.32 µm). The vessels relaxed in a dose-dependent manner and 100% relaxation was achieved with 10^−3^ mol/L SNP (Figure 6A).

Isolated DVR were subjected to hypoxia or normoxia for 30 min in the presence of 10^−4^ mol/L L-NAME to inhibit cellular NOS, followed by pre-constriction with 10^−6^ mol/L Ang II. Both groups of vessels had comparable diameters after pre-constriction (mean ± SEM: 2.72 ± 0.82 µm (hypoxia) vs. 2.51 ± 0.53 µm (normoxia), Mann–Whitney test, *p* > 0.05). A bolus of 10^−3^ mol/L SNP was then applied to these vessels to study the effect of hypoxia on the NO-dependent activation of sGC. Both groups of vessels showed similar relaxation in response to the bolus over a period of 10 min (Figure 6B). The effect of the NO-independent activation of sGC was similarly analysed by applying a bolus of 10^−6^ mol/L BAY 60-2770 to L-NAME-treated hypoxic and normoxic vessels that were pre-constricted using 10^−6^ mol/L Ang II. Both groups of vessels showed similar relaxation in response to BAY 60-2770 over a period of 10 min (Figure 6C). The absolute initial diameters of hypoxic vessels after pre-constriction (mean ± SEM: 1.70 ± 0.19 µm) were smaller compared to normoxic vessels (2.47 ± 0.24 µm, Mann–Whitney test, *p* < 0.05). However, the NO-independent activator BAY 60-2770 caused a significantly quicker relaxation in L-NAME-treated pre-constricted hypoxic vessels compared to the NO donor SNP (Figure 6D). The absolute initial diameters of pre-constricted hypoxic vessels treated with BAY 60-2770 (mean ± SEM: 1.70 ± 0.19 µm) were not significantly different than the SNP-treated vessels (2.72 ± 0.82 µm, Mann–Whitney test, *p* > 0.05).

## 3. Discussion

In this study, we analysed the function of isolated, perfused outer medullary DVR to demonstrate the importance of the NO system for vascular tone. Under physiological conditions, DVR constricted strongly in response to Ang II and relaxed completely in response to the ACh treatment that followed. Moreover, the dilatory function of DVR could be substantially enhanced by pharmacological modulation of the NO system, as evident from their strong dilatory responses to the PDE5 inhibitor sildenafil and the sGC activator BAY 60-2770. After exposure to a strong and acute hypoxia, DVR response to Ang II showed a significant increase, while there was a reduction in ACh-mediated dilation. This corresponds to the imbalance between vasoconstriction and dilation that leads to reduced renal (medullary) perfusion seen in ischemia/reperfusion models of AKI. Interestingly, the natural agonist of sGC, NO, as well as the sGC activator could dilate DVR after H/R, but sildenafil could not. Although all of these pharmacological agents increase cellular cGMP levels, their ability to do so seems to be differently affected by H/R.

We used isolated, perfused DVR to characterize the NO-sGC-cGMP system and to test the dilatory potency of pharmacological substances. This method is rarely applied due to its technically demanding nature that necessitates long-term training. Nevertheless, it is a well-established method and has been used in functional, electrophysiological, and imaging studies [25,26]. It has several advantages compared to the living kidney slice technique. For instance, while living kidney slices suffer from a lack of oxygen in the inner parts owing to their commonly used thickness of 200–300 µm, isolated, perfused DVR allow for sufficient oxygenation. This lack of oxygen in the slices may lead to metabolic changes in the tubuli and vessels, resulting in a release of a cocktail of substances with potentially vasoactive properties. In the case of isolated, perfused DVR, however, the experimental conditions can be uniformly controlled with the help of the bath and perfusion solutions. They can also be easily exposed to hypoxia and re-oxygenated in a precise and controlled fashion. Moreover, the perfusion also closely simulates physiological conditions as the flow itself is an important determinant of endothelial function.

The responses of isolated, perfused rat DVR to Ang II and ACh in our experiments were consistent with previously published studies [27,28,29]. The inhibition of NOS clearly enhanced Ang II response and diminished ACh-induced dilation, suggesting that NO is an important regulator of DVR diameter. NO activates sGC in vascular smooth muscle cells and pericytes, leading to cGMP production. This cGMP then activates protein kinase G, which phosphorylates several proteins that reduce the levels of cytosolic calcium, which in turn causes vasodilation [30].

While NO is the natural agonist of sGC, pharmacological agents can activate sGC independently of NO. These sGC activators are functional with both oxidized and haem-free variants of sGC [22]. We tested the sGC activator BAY 60-2770 in DVR, in which NO was depleted using L-NAME. BAY 60-2770 was indeed able to dilate pre-constricted DVR in a dose-dependent manner. This observation indicates that BAY 60-2770 may have a high potency to dilate NO-deficient DVR in vivo. Cinaciguat, another sGC activator, has also been shown to normalize renal resistance and blood flow in rats after L-NAME treatment [31]. However, activators are considered to exert a systemic action, which may reduce the overall arterial blood pressure and reverse the intended restoration of renal perfusion in pathological situations [31,32]. We also tested BAY 60-2770 on *human* DVR; however, these tests were without L-NAME pre-treatment due to the limited time available for acute experiments after harvesting the tissue and its subsequent transport to the laboratory. Nevertheless, BAY 60-2770 very effectively dilated pre-constricted human DVR and has potential for clinical application. In addition to sGC, cGMP levels are also regulated by PDEs. Here, we showed that PDE5 is an important component of the NO-sGC-cGMP system, and its inhibition had a strong dilatory effect on DVR under physiological conditions.

After characterizing the NO-sGC-cGMP system, we tested the ability of sildenafil, BAY 60-2770, and the NO donor SNP to dilate DVR after H/R. In most models of renal pathologies, including AKI and CKD, H/R is a major contributor to the pathogenesis of renal damage [33]. Animal models of ischemia/re-perfusion injury show reduced oxygenation and perfusion of the kidney. Furthermore, the restoration of blood flow and oxygenation after ischaemia is remarkably delayed in the renal medulla compared to the cortex [34,35]. This delay does not only indicate that the regulation of medullary perfusion after ischaemia is at least partly independent of that of the cortex, but also underscores the critical role that DVR play in it. Likely reasons for this medullary malperfusion could be a combination of functional changes such as the thrombotic occlusion of microvessels and an increased DVR tone [34]. The latter seems to be caused by an imbalance between vasoconstrictors and dilators. An increase in NO production and a reduced response to Ang II, as seen in ex vivo functional experiments in rat DVR, 48 h after warm renal ischaemia/re-perfusion, can be interpreted as a compensatory reaction to this imbalance. A rise in iNOS expression may trigger the increase in NO-bioavailability [26]. In kidney slices, fixed immediately following acute H/R (1 h each), vasa recta have been shown to have reduced diameters at pericyte sites and disruptions in their fluorescent dye-filled lumina [34]. Interestingly, the diameters of isolated DVR in the present study after 30 min of hypoxia followed by 10 min of re-oxygenation did not differ significantly from those of normoxic controls in the absence of vasoactive substances. However, the vessel response to Ang II was stronger, and ACh-induced dilation was weaker. Ang II activates NO synthase via Ang II receptor type I, resulting in NO release. This NO then dampens the Ang II-induced vasoconstriction in renal microvessels [36]. This crosstalk between Ang II and the NO system may be impaired after H/R, contributing to the stronger Ang II response and diminished ACh response. Another important factor that comes into play in this context is oxidative stress. Superoxide, a prominent representative of reactive oxygen species, does not only increase the Ang II response, since it is a part of the signalling, but also scavenges NO at the same time [37,38,39]. A similar increase in the Ang II response of DVR after H/R has also been observed in living kidney slices [40]. Taken together, functional changes in the outer medullary DVR seem to play a critical role in the disruption of medullary perfusion caused by ischaemia/re-perfusion.

Since the acute period is characterized by an increased tone and reactivity to Ang II, accompanied by reduced dilatory capacity, restoring vasodilation would be protective for the kidney. The NO donor SNP showed a full dilatory potency. This was unexpected because increased ROS generation after H/R may oxidize sGC, thereby rendering it less responsive to NO [37,41]. The NO-independent activation of sGC using BAY 60-2770 also led to complete DVR dilation, which was faster than the SNP-induced dilation. Surprisingly, sildenafil did not affect the DVR diameter after H/R at all, which may at least partly be due to low cGMP levels, as indicated by the significantly reduced response to ACh. However, direct damage to the enzyme due to the strong hypoxia cannot be ruled out.

Our findings suggest a beneficial effect of NO donors and sGC activators in hypoxia-damaged DVR in an acute pathological situation, where dilation is reduced and reactivity to Ang II is increased. While the period of re-oxygenation was relatively short in our experiments, prolonged re-oxygenation periods following strong hypoxia have also been shown to increase Ang II response in cortical microvessels in living kidney slices [42]. Therefore, one can speculate that longer re-oxygenation periods in vivo induce oxidative stress, resulting in stronger oxidation of sGC, making it unfit to be activated by NO. In such a situation, sGC activators are especially beneficial as they activate oxidized sGC more efficiently [43]. Therefore, the effect of sGC activators might be more pronounced in kidneys damaged by ischaemia/reperfusion, suggesting potential for therapeutic application.

## 4. Materials and Methods

### 4.1. Experimental Animals

Male Sprague Dawley rats were maintained at the animal facility of the Charité—Universitätsmedizin Berlin under a 12 h light/dark cycle. They were housed in enriched cages and were allowed free access to rat chow and tap water.

### 4.2. Dissection of DVR

To isolate DVR, rats (250 g) were anesthetized with isoflurane and then decapitated. The left kidney was then taken out immediately and sliced along the corticomedullary axis. A customized set of forceps (No. 5, Dumont, Switzerland) was used to isolate DVR from the renal outer medulla. A single DVR was then transferred to a perfusion chamber assembled on the stage of an inverted microscope. For some of the experiments, small bundles of DVR were dissected and pre-treated, e.g., in a hypoxic chamber, so that they could be easily retrieved after the pre-treatment to isolate single DVR for perfusion experiments. To follow the 3R principle of ‘reduce’, multiple DVR were isolated from each animal; however, no more than one DVR per animal was used for the same experimental protocol. Dulbecco’s modified Eagle’s medium (DMEM, Gibco, Paisley, UK) with 0.1% albumin (Carl Roth GmbH, Karlsruhe, Germany) was used as a bath solution for dissections as well as in the perfusion chamber.

### 4.3. Human DVR

Human DVR were isolated from non-malignant outer medullary renal tissue. The tissue was obtained from 6 patients who underwent nephrectomies due to renal cell carcinoma at the Klinik für Urologie, Charité—Universitätsmedizin Berlin between October 2019 and March 2022. All patients provided written informed consent. The study was approved by the ethical committee of the Charité—Universitätsmedizin Berlin (Approval No. EA4/65/18).

### 4.4. Perfusion of Isolated DVR

**Figure 7:**
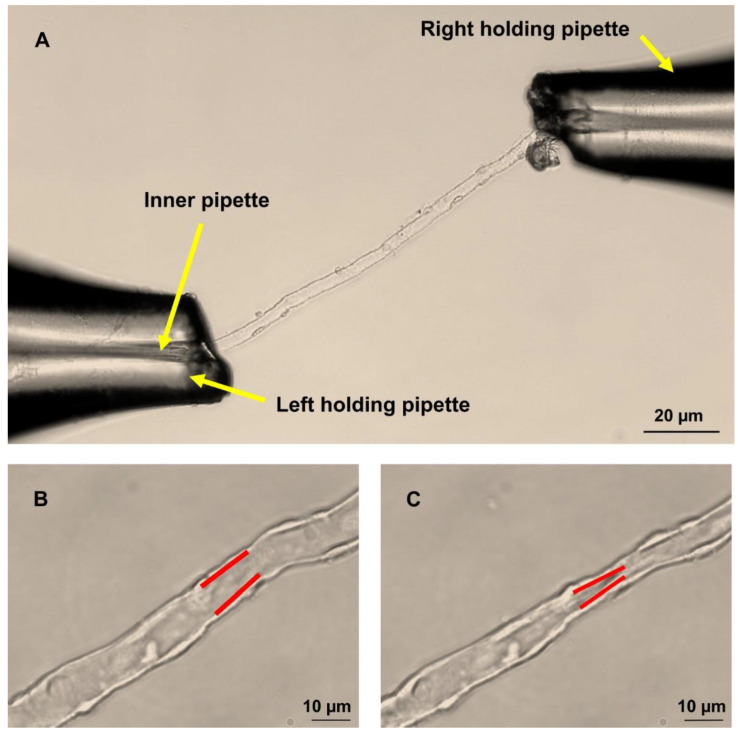
(**A**) Microperfusion of rat DVR using holding and perfusion pipettes. (**B**) Resting and (**C**) constricted DVR with the site of constriction marked in red.

A set of handmade glass pipettes were used to perfuse the DVR. In the perfusion chamber, a single DVR was fixed in place using a holding pipette on each end. A smaller pipette placed inside the left holding pipette (inner pipette) was advanced into the lumen of the vessel (Figure 7A). The vessel was then perfused with DMEM supplemented with 1% albumin. The perfusion was carried out under a pressure of 15 mm Hg using a pressure head. This pressure is suitable to open the lumen of the DVR without any sign of overstretching. After warming to 37 °C, vessels were allowed to adapt for 5 min before starting the experiment. All experiments were performed within 2 h after the animals were sacrificed.

### 4.5. Measurement of DVR Diameters

During the experiments, vessels were continuously displayed on a computer screen using a video camera (Moticam 2.0, Motic Asia, Hong Kong, China). Luminal diameters served for the estimation of vascular tone and reactivity and were measured using the freeware ImageJ at the site where the reaction to the agonist being tested was the strongest [44]. DVR do not react to agonists uniformly across their length since pericytes, their vasoactive parts, do not completely cover their outer surface (Figure 7B,C). To analyse the effects of pre-treatments and for concentration–response curves, an image was taken every second and average vessel diameters were calculated using measurements from five consecutive images. For time–response curves, diameters were measured from single images taken every 10 s over a period of 10 min.

### 4.6. Protocols

All chemicals and drugs were purchased from Sigma-Aldrich (Darmstadt, Germany), unless otherwise specified. Stock solutions of substances insoluble in distilled water were prepared in dimethyl sulfoxide (DMSO, purity > 99.7%, Bellefonte, PA, USA). The final concentration of DMSO did not exceed 0.1% in any of the experiments. All chemicals were stored at −20 °C. Concentrations are given as final molar concentration in the bath solution.

#### 4.6.1. Pharmacological Characterization of NO-sGC System

To test the contribution of the NO system to the DVR tone, vessels were incubated in bath solution with or without L-NAME (10^−4^ mol/L) for 15 min. Then, Ang II was given in increasing concentrations (10^−12^ to 10^−6^ mol/L, 2 min each). After reaching a stable constriction, ACh was applied in cumulatively increasing concentrations (10^−11^ to 10^−4^ mol/L, 3 min each).

The effect of PDE5 inhibition on DVR was tested using sildenafil (Biomol GmbH, Hamburg, Germany). Isolated rat DVR were pre-constricted using 10^−6^ mol/L Ang II followed by treatment with increasing concentrations of sildenafil (10^−9^−10^−6^ mol/L, 3 min each) to obtain the concentration–response curves. The dynamics of vessel dilation were investigated by applying a bolus of sildenafil (10^−7^ mol/L) or a corresponding amount of DMSO (solvent control) and tracking the changes in vessel diameters over a period of 10 min.

To study the effect of sGC activation, isolated rat DVR were pre-treated with 10^−4^ mol/L L-NAME for 15 min followed by pre-constriction with 10^−6^ mol/L Ang II. A concentration–response curve was then obtained by applying the sGC activator BAY 60-2770 (Bayer AG, Wuppertal, Germany) in cumulatively increasing concentrations (10^−11^ to 10^−5^ mol/L, 3 min each). To obtain the time–response curve, L-NAME-treated Ang II pre-constricted DVR were treated with a bolus of 10^−6^ mol/L BAY 60-2770 or a corresponding amount of DMSO (solvent control) and the changes in vessel diameters were tracked over a period of 10 min.

#### 4.6.2. Human DVR

To check vessel viability and simultaneously pre-constrict the DVR, Ang II was applied in increasing concentrations (10^−12^ to 10^−6^ mol/L, 2 min each). After reaching a stable constriction, BAY 60-2770 (10^−6^ mol/L) was applied and the changes in the diameter were tracked over a period of 10 min.

#### 4.6.3. Effect of Hypoxia on the NO System

To investigate how hypoxia influences the NO system, DVR were incubated in an environment with either 0.1% O_2_ (hypoxia) or 20.9% O_2_ (normoxia) for 30 min. Hypoxic conditions were achieved using a hypoxia chamber (H35 hypoxystation, Don Whitley Scientific Ltd., West Yorkshire, UK). After a re-oxygenation period of 10 min, Ang II concentration–responses (10^−12^ to 10^−6^ mol/L, 2 min each) were measured. In an additional series of experiments, ACh was applied (10^−11^ to 10^−4^ mol/L, 3 min each) after pre-constriction with Ang II (10^−6^ mol/L) to obtain the concentration–response for ACh in normoxic and hypoxic DVR. Sildenafil was applied as bolus (10^−7^ mol/L) after H/R and pre-constriction with Ang II (10^−6^ mol/L). Changes in vessel diameters were tracked over a period of 10 min.

The NO donor SNP was used to test the natural stimulation of sGC. The concentration–response was measured after L-NAME (10^−4^ mol/L) pre-treatment and Ang II (10^−6^ mol/L) pre-constriction. Furthermore, the time response to bolus application of SNP (10^−3^ mol/L) was measured in normoxic and hypoxic DVR. Time–responses to the sGC activator BAY 60-2770 were measured after Ang II (10^−6^ mol/L) pre-constriction and L-NAME (10^−4^ mol/L) pre-treatment in DVR after H/R or normoxia.

### 4.7. Statistics

Mean and standard error of the mean (SEM) were calculated using GraphPad Prism 9.3.1 (GraphPad software, San Diego, CA, USA). Data were tested for normal distribution using the Shapiro–Wilk test. Although most data were normally distributed, we used nonparametric statistical tests in this study as they provide the most robust testing. Differences between concentration- or time-dependent changes in vascular diameters were tested by Brunner test for repeated measurements, provided by the “R” project, which is a nonparametric counterpart of the two-way ANOVA [45]. Differences between initial diameters were tested by using the Mann–Whitney test for independent measurements. The effect of L-NAME on vascular diameters was tested using the Wilcoxon test for dependent measurements (GraphPad Prism 9.3.1). Differences were assumed to be significant if *p*-values were <0.05.

## Data Availability

The data presented in the study are contained within the article.
